# Effect of acupoint catgut embedding in chronic fatigue syndrome patients: A protocol for systematic review and meta-analysis

**DOI:** 10.1097/MD.0000000000023946

**Published:** 2021-02-05

**Authors:** Mei-Lin Zhang, Hong-Juan Fu, Yong Tang, Zhen-Guo Luo, Jian-Yong Li, Rui Li

**Affiliations:** a Acupuncture-moxibustion School of Affiliated Hospital of Chengdu University of Traditional Chinese Medicine; b School of Acupuncture-moxibustion and Tuina, Chengdu University of Traditional Chinese Medicine; c Sichuan Integrative Medicine Hospital, Chengdu, Sichuan, China.

**Keywords:** acupoint catgut embedding, chronic fatigue syndrome, meta-analysis, protocol, systematic review

## Abstract

**Background:**

Chronic fatigue syndrome (CFS) is a relatively complex and disabling illness with a substantial economic burden and functional impairment. Until now, many CFS patients lack appropriate healthcare. Acupoint catgut embedding is an effective and emerging alternative therapy for CFE. With this research, we endeavor to investigate the effect and safety of ACE for CFS.

**Methods:**

Eight databases will be searched from inception to December 2020: PubMed, EMBASE, The Cochrane Library, Web of Science, China National Knowledge Infrastructure, Chinese Biomedical Literature Database, Chong-Qing VIP database, and Wan-fang database. We regard studies as eligible for inclusion if they were RCTs done in CFS patients, compare acupoint catgut embedding to another treatment strategy, and report fatigue changes at the end of the intervention period. Two independent reviewers complete the study selection, data extraction, and the risk of bias assessment. We assess pooled data using a random-effects model through Revman software (v.5.3) and Stata (version 15.0).

**Ethics and dissemination:**

Ethics approval is not required because the individual patient data will not be involved, with no privacy concerns. This systematic review and meta-analysis will provide a reference for CFS patients and clinicians on the non-drug interventions. We will publish and disseminate the results of this review in a peer-reviewed journal or relevant conference.

**OSF Registration number:**

10.17605/OSF.IO/7SHD9 (https://osf.io/7shd9).

## 1 Introduction

Chronic fatigue syndrome (CFS) is a severe, debilitating condition that affects millions of people worldwide,^[1]^ causing profound, prolonged illness and disability.^[2–4]^ CFS is characterized by medically unexplained fatigue that persists for more than 6 months and comprises a range of symptoms, fluctuating in intensity and severity. Patients with CFS are more functionally impaired than those with other disabling illnesses, such as multiple sclerosis, systemic lupus erythematosus, rheumatoid arthritis, congestive heart failure, and other chronic conditions.^[2]^ It has been reported that at least one-quarter of CFS patients are at home or bed-ridden at some point in their lives,^[5]^ imposing an enormous burden for patients, their caregivers, the health care system, and society, with an estimated direct and indirect economic cost of $18 to $24 billion annually.^[6–10]^ The 3 most prevalent causes of death in CFS patients were heart failure, suicide, and cancer.^[11]^ CFS patients mean age of death is considerably younger than those who died from cancer and suicide in the general population.^[12]^

Although researchers pay substantial efforts to understand CFS better, diagnosing the disease is still a challenge because patients often struggle with their illness for years before an identification is made.^[1,2]^ Due to CFS diagnosis uncertainties and a lack of clinical guidance for clinicians, many patients do not receive appropriate healthcare.^[6]^ Research into CFS focus on treatment or management increasing only in recent years. At present, cognitive-behavioral therapy (CBT) is known as one commonly used in treating CFS, with evidence supporting its effectiveness and cost-effectiveness.^[13,14]^ However, it may be argued that currently there is no comprehensive agreement on what counts as “CBT”.^[15]^ To further improve effectiveness, researchers advocate investigating multidisciplinary treatments, including CBT in combination with other interventions, such as complementary alternative therapies.^[16]^

Acupoint catgut embedding (ACE) in traditional Chinese medicine (TCM) is an alternative treatment and comes to the attention of researchers.^[17]^ ACE involves the implantation of absorbable sutures at traditional Chinese meridian acupoints, with the dual effects of acupuncture and suture absorption stimulation.^[18]^ ACE is a combination product of ancient traditional acupuncture and modern tissue therapy,^[19]^ which can conserve time and costs compared to other alternative therapies.^[20,21]^ Currently, CFSs etiology involves various factors, including immunological, genetic, viral, neuroendocrine and psychological, especially being closely related to immunity and inflammation.^[22]^ This technique is supposed to be beneficial to relieve fatigue in CFS patients.^[23–25]^ Previous researches conformed that ACE exhibits analgesic effects,^[19,20,26]^ improves immune system function,^[23]^ and mitigates inflammation and oxidative stress.^[27]^ Besides, Yang^[28]^ founded that the effect of ACE for CFS is similar to ginsenoside. Therefore, acupoint catgut embedding has the potential to be a useful supplementary treatment option for chronic fatigue syndrome. However, ACEs effect on CFS is still controversial based on the current evidence-based medical evidence. Therefore, the present works chief aim is to obtain a relatively convincing conclusion of whether acupoint catgut embedding is useful for alleviating fatigue in patients with chronic fatigue syndrome.

## 2 Methods

### 2.1 Study registration

This protocol strictly follows the PRISMA-P guidelines^[29]^ with an Open Science Frame-work registration number 10.17605/OSF.IO/7SHD9 (https://osf.io/7shd9).

### 2.2 Eligibility criteria

#### 2.2.1 Types of studies

RCTs investigating the effect of acupoint catgut embedding (ACE) on relieving fatigue in CFS patients will be included.

#### 2.2.2 Types of participants

Participants diagnosed with CFS by either of the following diagnostic criteria will be included: Fukuda Case Definition for CFS (1994),^[30]^ Canadian Consensus Criteria for ME/CFS (2003), NICE Clinical Guidelines for CFS/ME (2007),^[2]^ Revised Canadian Consensus Criteria for ME/CFS (2010), International Consensus Criteria for ME (2011),^[31]^ and The Institute of Medicine (2015).^[1]^

#### 2.2.3 Types of interventions

Intervention measure is ACE, regardless of intervention duration, frequency, acupoint, and absorbable suture types. ACE and adjunct therapy combination treatment will be included.

#### 2.2.4 Types of comparators

The comparison groups involve another treatment strategy, such as cognitive-behavioral therapy (CBT), acupuncture, moxibustion, or sham ACE.

#### 2.2.5 Types of outcome measures

The primary outcome is fatigue outcomes scale: Fatigue Scale-14 (FS-14), Brief Fatigue Inventory (BFI), Fatigue Severity Scale (FSS), Checklist Individual Strength (CIS), and Fatigue Impact Scale (FIS). The secondary outcomes are quality of life, pain, insomnia, anxiety, and other clinical outcomes.

### 2.3 Search methods for identification of studies

#### 2.3.1 Information sources

Two researchers (YT and ZGL) independently select relevant studies published from inception to December 1, 2020 by searching PubMed, Embase, Web of Science, The Cochrane Library, Chinese National Knowledge Infrastructure (CNKI), Wanfang Database, the Chongqing VIP Chinese Science and Technology Periodical Database (VIP), Chinese Biomedical Literature Database (CBM), and ClinicalTrials.gov. We apply no language restrictions. Besides, we will manually search the reference list of critical articles to find potential studies.

#### 2.3.2 Search

We use the following combined text and MeSH terms: acupoint catgut embedding, catgut embedding, chronic fatigue syndrome, fatigue syndrome. An example of the search strategy for PubMed is in Table 1.

**Table 1:** Search strategy for PubMed.

#1	“acupoint catgut embedding” [Title/Abstract] OR “ catgut embedding ” [Title/Abstract] OR “ catgut ” [Title/Abstract]
#2	“Chronic Fatigue Syndrome” [MeSH Terms]
#3	“Chronic Fatigue Syndrome” [Title/Abstract] OR “fatigue” [Title/Abstract] OR “tired” [Title/Abstract] OR “weariness” [Title/Abstract] OR “weakness” [Title/Abstract] OR “asthenia” [Title/Abstract]
#4	#2 OR #3
#5	“Clinical Trials as Topic” [MeSH Terms]
#6	“Randomized Controlled Trials as Topic” [MeSH Terms]
#7	“randomized controlled trial” [Title/Abstract] OR “clinical trial” [Title/Abstract] OR “clinical study” [Title/Abstract] OR “randomized trial” [Title/Abstract] OR “controlled trial” [Title/Abstract]
#8	#5 OR #6 OR #7
#9	#1 AND #4 AND #8

### 2.4 Data collection and analysis

#### 2.4.1 Study selection

**Figure 1:**
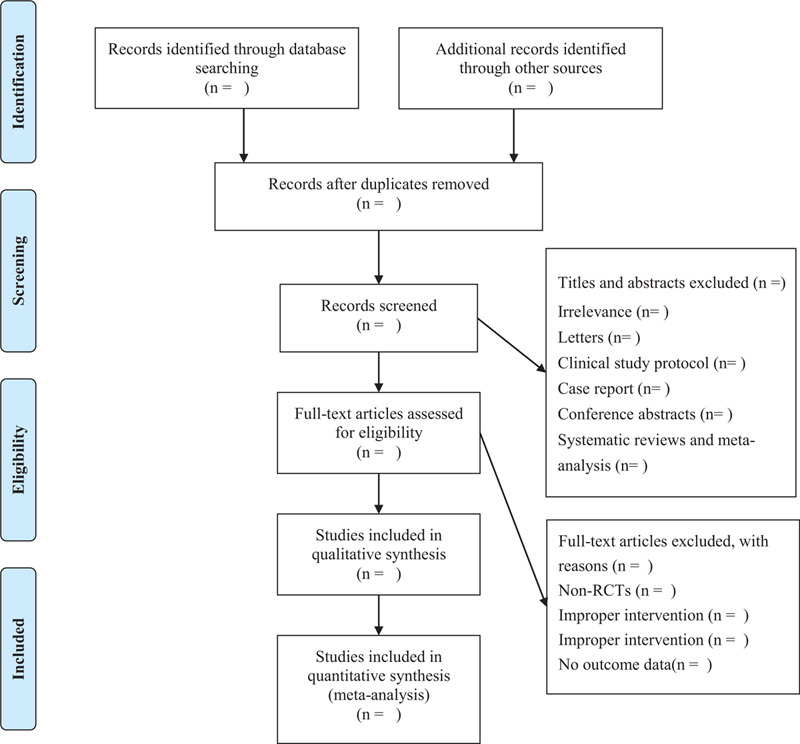
Flow chart of the study.

Two independent investigators (YT, JYL) will import search results into EndNote X8 (Bld 10063) and then remove duplicate articles. They review titles and abstracts to remove irrelevant literature, non-RCTs, animal experiments, case reports, and systematic reviews. Studies that satisfy the inclusion criteria will be retrieved for full-text assessment. The exclusion reasons will be recorded in an excel table. Disagreements will be resolved by a third reviewer (MLZ). The study selection process of this review will be presented in Figure 1.

#### 2.4.2 Data extraction

Two reviewers (ZGL, RL) independently extract the following data from each eligible study: the first author, year of publication, country of origin, number of participants, age, sex, diagnostic criteria, intervention duration, frequency, acupoints, absorbable suture types, changes in outcomes, adverse events at the end of the intervention. We will cross-check all data and transfer it into Review Manager (version 5.3, Cochrane Library) and Stata (version 15.0). Any disagreements will be arbitrated by a third reviewer (MLZ).

#### 2.4.3 Assessment of risk of bias

We apply The Cochrane Collaborations tool for assessing the risk of bias (RoB) in included RCTs according to the following 7 aspects: random sequence generation, allocation concealment, participant blinding, analyst blinding, data completeness of results, selective reports, and other sources of bias. Disagreements will be resolved by a third reviewer (MLZ).

#### 2.4.4 Summary measures

We will make a meta-analysis when the number of eligible studies was more than one in each outcome. We calculate the standardized mean difference (SMD) with a 95% confidence interval (CI) and select a random-effect model to pool data. For categorical outcomes, we calculate pooled estimates of the relative risk with a random-effects model. The Review Manager (version 5.3, Cochrane Library) and Stata (version 15.0) will be applied for statistical analyses.

#### 2.4.5 Unit of analysis issues

This review will not involve individual patient data. Different units of the outcome will be converted to international units before analysis.

#### 2.4.6 Dealing with missing data

The corresponding authors will be contacted by telephone or email to obtain the missing information. We will discuss the potential impact of missing data on the final findings of the review.

#### 2.4.7 Assessment of heterogeneity

This protocol adopts forest plots and *I*^*2*^ statistics to assess the magnitude of the heterogeneity between studies. *I*^*2*^ values of 0 implying no heterogeneity; values of 25% mean low heterogeneity; values greater than 50% are supposed to be indicative of moderate-to-high heterogeneity.^[32]^ We will explore the sources of heterogeneity when significant heterogeneity was detected.

### 2.5 Synthesis of results

We option the random-effects model to synthesis the data. The meta-analysis will not be conducted when considerable heterogeneity existed between studies, which is unexplainable, and a narrative summary will be presented.

#### 2.5.1 Risk of bias across studies

We will evaluate the possibility of publication bias by constructing a funnel plot when more than 10 studies are included. We also further assess funnel plot asymmetry using Begg and Egger tests and define significant publication bias as a *P* value <.1. The trim-and-fill computation will be applied for estimating the impact of publication bias on the interpretation of the results.^[33]^

#### 2.5.2 Subgroup analysis

We will perform subgroup analyses according to variations in the duration, frequency, and absorbable suture types of ACE when data are available.

#### 2.5.3 Sensitivity analysis

We will conduct a sensitivity analysis to monitor the robustness of the preliminary decision made in the review process. We also further evaluate the impact of a single study on the overall pooled estimate.

#### 2.5.4 Evidence quality evaluation

We will use the Grades of Recommendations Assessment, Development and Evaluation (GRADE) system to evaluate the certainty of merged data. The quality of evidence will be specified to 4 grades: high, moderate, low, and very low quality.^[34]^

## 3 Discussion

Previous researches have demonstrated that acupoint catgut embedding is beneficial for alleviating fatigue in CFS. Due to the small sample of studies, the traditional systematic assessment cannot reach an accurate conclusion on ACE intervention. We designed this protocol based on previous studies to throw light on the nature of ACEs effect and safety for CFS. This systematic review and meta-analysis will provide a relatively convincing conclusion of whether acupoint catgut embedding effectively treats chronic fatigue syndrome patients. Conclusions drawn from this review may benefit patients, clinical practitioners, and policymakers.

## Author contributions

**Conceptualization:** Mei-Lin Zhang.

**Data curation:** Yong Tang, Zhen-Guo Luo.

**Formal analysis:** Yong Tang, Jian-Yong Li.

**Funding acquisition:** Mei-Lin Zhang.

**Resources:** Mei-Lin Zhang.

**Software:** Rui Li.

**Writing – original draft:** Mei-Lin Zhang.

**Writing – review & editing:** Hong-Juan Fu.
